# Specificity in the Susceptibilities of *Escherichia coli*, *Pseudomonas aeruginosa* and *Staphylococcus aureus* Clinical Isolates to Six Metal Antimicrobials

**DOI:** 10.3390/antibiotics8020051

**Published:** 2019-05-01

**Authors:** Natalie Gugala, Dennis Vu, Michael D. Parkins, Raymond J. Turner

**Affiliations:** 1 Department of Biological Sciences, University of Calgary, Calgary, AB T2N 1N4, Canada; ngugala@ucalgary.ca (N.G.); devu@ucalgary.ca (D.V.); 2 Cumming School of Medicine, University of Calgary, Calgary, AB T2N 1N4, Canada; mdparkin@ucalgary.ca

**Keywords:** metals, metal antimicrobials, *Escherichia coli*, *Staphylococcus aureus*, *Pseudomonas aeruginosa*, metal ions

## Abstract

In response to the occurrence of antibiotic resistance, there has been rapid developments in the field of metal-based antimicrobials. Although it is largely assumed that metals provide broad-spectrum microbial efficacy, studies have shown that this is not always the case. Therefore, in this study, we compared the susceptibilities of 93 clinical isolates belonging to the species *Escherichia coli*, *Pseudomonas aeruginosa* and *Staphylococcus aureus* against six metals, namely aluminum, copper, gallium, nickel, silver and zinc. To provide qualitative comparative information, the resulting zones of growth inhibition were compared to the minimal inhibitory concentrations of three indicator strains *E*. *coli* ATCC 25922, *P. aeruginosa* ATCC 27853 and *S. aureus* ATCC 25923. Here, we demonstrate that the metal efficacies were species- and isolate-specific. Only several isolates were either resistant or sensitive to all of the six metals, with great variability found between isolates. However, the greatest degree of similarity was found with the *E*. *coli* isolates. In contrast, the susceptibilities of the remaining two collections, *S. aureus* and *P. aeruginosa*, were more highly dispersed. Using this information, we have shown that metals are not equal in their efficacies. Hence, their use should be tailored against a particular microorganism and care should be taken to ensure the use of the correct concentration.

## 1. Introduction

In our modern era, the rising incidence of antibiotic resistance is a familiar concern that continues to provide challenges in infection control and disease prevention [1,2]. In response, in the last several decades, we have seen an increase in the development of alterative antimicrobials, including peptides [3] and polymers [4] and modifications to traditional therapeutic regimes, such as combination treatments [5]. Metals and metal-based antimicrobials are among these alternative agents that are presently being investigated (see review [6] for more information).

As their name suggests, essential metals, such as zinc, copper and iron, are essential to the biochemistry of life. It has been estimated that at least one-third of all proteins require metals [7,8,9]. Despite this, elevated concentrations of these proteins can cause microbial toxicity. Non-essential metals, including silver, gallium and tellurium, result in a similar fate but at considerably lower concentrations [10,11]. Presently, advancements in the biomedical applications of metals primarily take the form of diagnostic procedures and the prevention of diseases following the discovery that metals can disrupt antibiotic resistant biofilms [11,12,13,14] and kill multidrug resistant bacteria [15,16,17,18] at low concentrations. For example, metals are now being impregnated into textiles, including socks and wound bandages [19,20]; coated onto surfaces, such as medical devices [21,22,23]; and incorporated into liquid formulations [24]. Metal-based antimicrobials, such as nanoparticles, generally highlighted for their elevated toxicity, are seeing an increase in development and use [15,25,26,27]. A number of metal infused hydrogels and polymers, which provide slow and concentrated release, have been developed and tested against microorganisms [28,29]. Moreover, metals are being combined with existing antimicrobials, such as antibiotics, as a means of improving their efficacy and repurposing agents that are no longer useful against multidrug resistant bacteria [30,31].

Metals and metal-based antimicrobials target shared biomolecules and are thus generally regarded as being broad-spectrum [32]. However, a number of studies have shown that metal ions [13,33], similar to metal nanoparticles [34,35] are not equivalent in their toxicity towards different strains. This is problematic, particularly since metal-based antimicrobials are being used in consumer products, such as activewear, deodorant and washing machines [36]. There is now strong evidence that metal resistance exists [37,38,39,40,41,42,43] and this increase is likely to drive antibiotic resistance further [44,45,46,47,48] since the mechanisms of antibiotics resistance, such as reduced toxin import, drug inactivation and mutation of toxin targets among others, are also common mechanisms of metal resistance (refer to review [46] for more detailed information). As a result, it is imperative that the precise toxicity of metals against microorganisms is identified to ensure that the correct concentrations of metal ions are utilized against the appropriate organism.

In this study, the antimicrobial efficacies of six metals, namely aluminum, copper, gallium, nickel, silver and zinc, were tested against 34 *Staphylococcus aureus*, 27 *Pseudomonas aeruginosa* and 32 *Escherichia coli* clinical isolates using the disk diffusion assay. The results were compared to the minimal inhibitory concentrations of the corresponding indicator strains *S*. *aureus* ATCC 25923, *P*. *aeruginosa* ATCC 27853 and *E*. *coli* ATCC 25922 in order to normalize and provide context to the zones of growth inhibition measured. We found the efficacies of the metals to be strain- and isolate-specific. The *E. coli* collection revealed the greatest degree of similarity although disparities were observed between a number of isolates. There were great differences in the susceptibilities of the *S. aureus* and *P. aeruginosa* isolates to aluminum, copper, gallium and silver and these observations were variable. Silver displayed the greatest efficacy, followed by aluminum and gallium. In contrast, the least efficacious metal was nickel. In this work, we demonstrated that metals are not equivalent in their antimicrobial abilities and isolates of the same species have varying susceptibilities. As a result, the use of metal-based antimicrobials should be tailored to a specific organism at a precise concentration.

## 2. Results

The efficacies of six metals, namely aluminum, copper, gallium, nickel, silver and zinc, were tested against 93 bacterial isolates using the disk diffusion assay, which allows for high-throughput susceptibility testing. In order to account for independent variables and provide reference, the zones of growth inhibition were normalized against the three pathogenic indicator strains, *S*. *aureus* ATCC 25923, *P*. *aeruginosa* ATCC 27853 and *E*. *coli* ATCC 25922, for which the MICs in chemically simulated wound fluid (CSWF) are known (Appendix A, Table A1). Since the MICs of the indicator strains were known, a qualitative concentration—to which we refer to as the breakpoint value—can be given to each isolate. A score of 1.0 is equal to the MIC of the reference indicator strain under the given metal stress. In contrast, a score <1.0 means that the breakpoint value is >MIC and a score >1.0 means that the breakpoint value <MIC (see Section 4—Materials and Methods).

**Figure 1:**
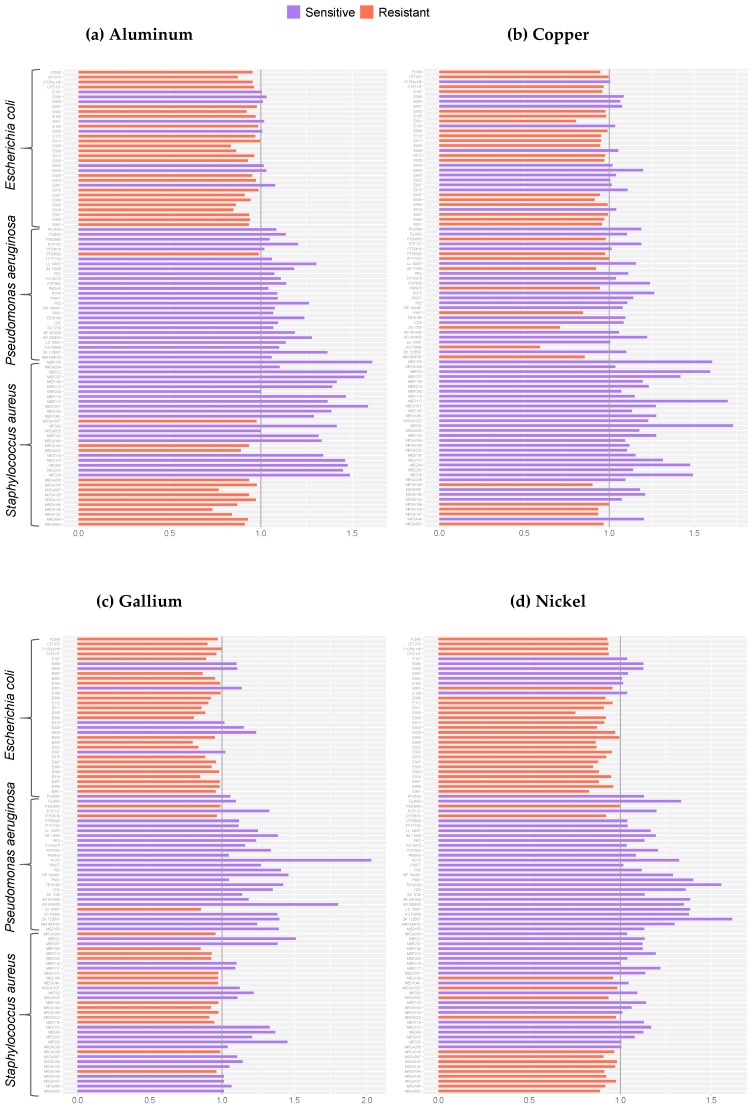

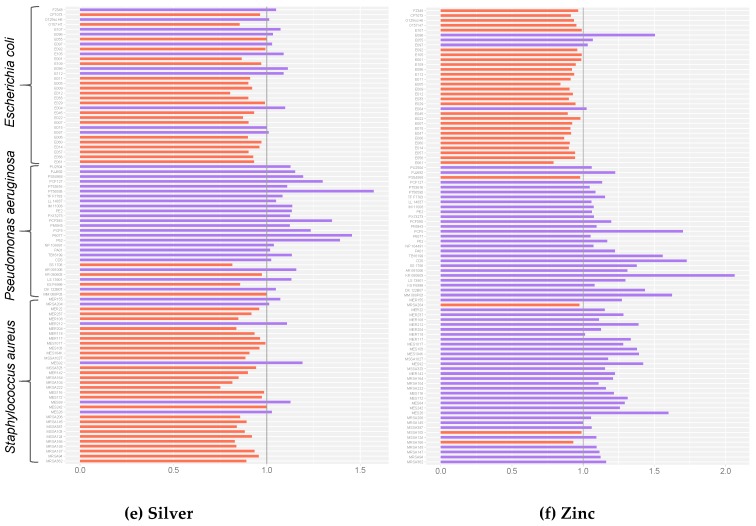
Bar plots signifying the normalized score for each isolate against the corresponding indicator strain, for which the value is 1.0 (grey line). This score represents the MIC of the indicator strain under the given metal stress. Orange denotes a resistant isolate. For these isolates, the zone of growth inhibition was less than the corresponding indicator strain (<1.0). Purple represents the isolates that fall above the normalized score since the zones of growth inhibition were larger. These are noted as sensitive isolates (>1.0). The scores represent the mean of three biological trials, with each having two technical replicates. The MICs have the following order: *E*. *coli* ATCC 25922, *P*. *aeruginosa* ATCC 27853 and *S*. *aureus* ATCC 25923: (**a**) aluminum: 250 mM, 1.95 mM and >250 mM; (**b**) copper: 12.5 mM, 6.25 mM and 12.5 mM; (**c**) gallium: 31.25 mM, 15.63 mM, 15.62 mM; (**d**) nickel: >625 mM, >650 mM and >625 mM; (**e**) silver: >0.5 mM, >0.5 mM and >0.5 mM; and (**f**) zinc: >650 mM, >375 mM and 23.44 mM.

Less variability between the *E. coli* isolates was observed when compared to the *P. aeruginosa* and *S. aureus* collections (Figure 1a–f). The three *E*. *coli* isolates, namely CFTO73, O127:H6 and O157:H7 (the latter noted as multidrug resistant [MDR] [49]), displayed resistance to all the metals except silver. When examining the *E. coli* collection in more detail, there were a number of isolates, including E009, E011, E012 and E056, that presented scores distant from the normalized score when grown in the presence of gallium, nickel, silver and zinc, respectively (Figure 1c–f). The scores of these isolates were below 1.0. Therefore, the breakpoint values were >31.25 mM, >625 mM, >0.50 mM and >650 mM, respectively (Supplementary Materials, Table S1). It is important to note that Supplementary Materials, Tables S1–S3 report the average diameters and the standard deviations in order to show the variability in the data set. Therefore, our reasoning for normalizing the values is justified. Figure 1 is not entirely comparable to Supplementary Materials, Tables S1–S3.

In general, the *P. aeruginosa* isolates were sensitive to all six metals. Several isolates were found to have scores that were two-fold higher than the normalized score of the indicator strain, such as PCF5 under gallium exposure; TB161 and DK122B07 under nickel exposure; PT56593 under silver exposure; and KR080603 under zinc exposure. The sensitivities of these isolates were pronounced in the presence of these metals but not with the other metal antimicrobials. Gallium was found to be efficacious against the *P. aeruginosa* isolates although this metal demonstrated variable efficacy against the *S. aureus* and *E. coli* isolates (Figure 1c). Within the concentrations tested, the efficacy of silver was the greatest against the *Pseudomonas* collection (Figure 1e). All but three isolates had breakpoints values that were <0.50 mM (Supplementary Materials, Table S2). 

In the presence of aluminum and copper, a number of the *S. aureus* isolates, such as MER155, ME101T and MES92, presented scores that were nearly 1.5-fold greater than *S. aureus* ATCC 25923 (Figure 1a,b). The breakpoint values were >250 mM and >12.50 mM, respectively (Supplementary Materials, Table S3). This trend was not met by the other metals. In fact, many of the aforementioned isolates were resistant to the concentration of silver tested in this study. Nearly all the MRSA (methicillin resistant) and MSSA (methicillin sensitive) isolates were resistant to aluminum, nickel and silver. Thus, the breakpoint values were >250 mM, >625 mM and >125 mM, respectively (Supplementary Materials, Table S3). 

**Figure 2:**
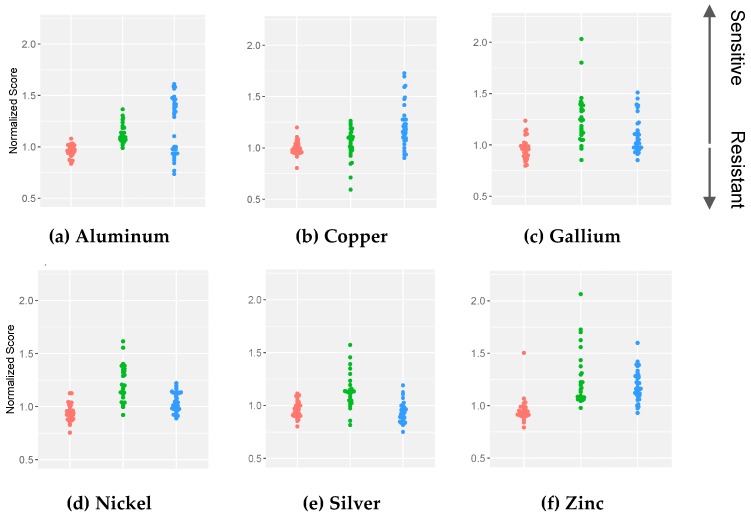
Dot plots illustrating the dispersity between the normalized scores of the *E. coli* (red), *P. aeruginosa* (green) and *S. aureus* (blue) isolates; (**a**) aluminum; (**b**) copper; (**c**) gallium; (**d**) nickel; (**e**) silver; and (**f**) zinc. The zones of growth inhibition for the isolates were normalized against the zones of the indicator strains. A value of 1.0 signifies the minimal inhibitory concentration corresponding to the indicator strain. Isolates with scores >1.0 were considered sensitive and those with scores <1.0 were noted as resistant. Each score represents the mean of three biological trials, with each having two technical replicates.

**Figure 3:**
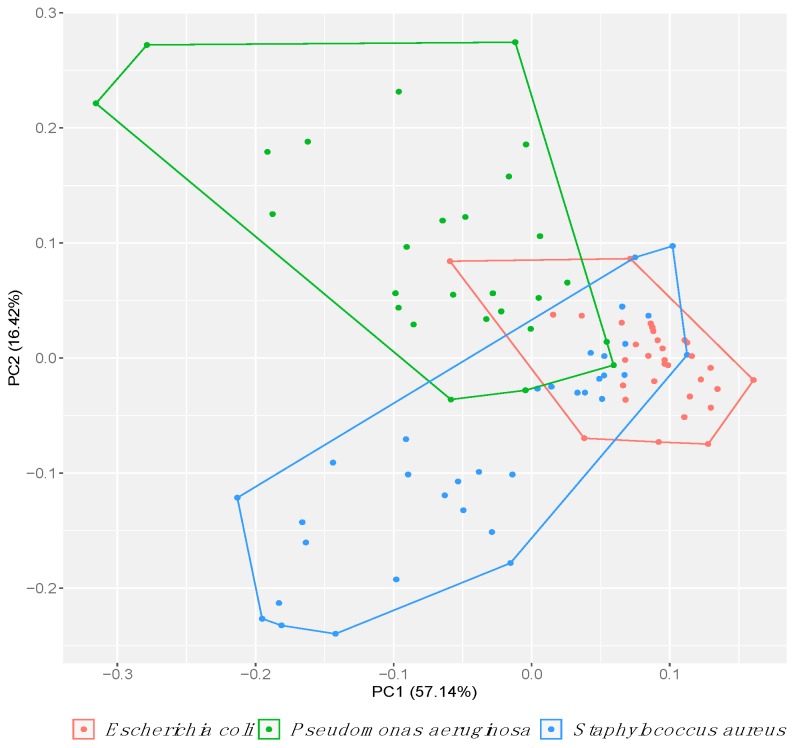
Clustering of the 93 isolates belonging to the *E. coli* (red), *P. aeruginosa* (green) or *S. aureus* (blue) species using principle component analysis. Collections were highlighted to show positioning of each isolate with respect to the remaining collection. Each isolate was normalized against the corresponding indicator strain in the presence of the six metals, namely aluminum, copper, gallium, nickel, silver and zinc. Data were collected from the mean of three biological trials, with each having two replicates.

The clustering of *Escherichia* and the dispersity of *Pseudomonas* and *Staphylococcus* are shown in Figure 2 and Figure 3. These plots demonstrate that the susceptibilities of the *P. aeruginosa* isolates were more dispersed than the *S. aureus* isolates in the presence of aluminum, a trend that was inverted for the metal gallium (Figure 2a,c). However, the overall spread of the two species is the same in the presence of copper. Upon comparison between the overall scatterings of the three species, the scores of the *Pseudomonas* collection dispersed to a greater degree (Figure 3). When considering all six metals together, the *E. coli* isolates clustered closely although this did not occur for the *P. aeruginosa* and *S. aureus* isolates (Figure 3).

Overall, all the isolates varied in their sensitivity to the six metals (Figure 3). However, the working metal solutions in this study were not equal. To account for these differences, the scores were normalized against the respective concentrations (Supplementary Materials, Figure S1). Here, the metals can only be compared to each other. Regardless, the overall trends between the isolates of a given metal remain the same. In general, the scores were found to be the highest for the metal silver, followed by aluminum and then gallium. In contrast, the metal nickel had the lowest scores (Supplementary Materials, Figure S1).

## 3. Discussion

In this study, the efficacies of six metals, namely aluminum, copper, gallium, nickel, silver and zinc, against 93 bacterial isolates were compared using the disk diffusion assay. To our knowledge, no breakpoint values have been reported for the three indicator strains, *E*. *coli* ATCC 25922, *P*. *aeruginosa* ATCC 27853 and *S*. *aureus* ATCC 25923, under a metal challenge. Using a preceding study completed by our group, the same MICs were used to provide breakpoint values. A rich growth medium, chemically simulated wound fluid (CSWF), containing bovine serum albumin among other components was used to simulate a wound environment. Finally, the zones of growth inhibition were measured, normalized and compared to the indicator strains.

Metal antimicrobials are generally regarded as broad-spectrum [6]. Nonetheless, studies have demonstrated that this is not always the case. We have shown that metal antimicrobials vary in their efficacy against different species and different isolates of the same species, with these differences not being uniform. For example, the isolate with the highest gallium sensitivity was *P. aeruginosa* KR080603 although this microorganism presented substantially less copper sensitivity compared to the remaining *P. aeruginosa* isolates. In fact, the most sensitive copper isolate was MES192, a *S. aureus* isolate. If metals behaved similarly in the presence of different microorganisms and their mechanisms of action were similar or the same, a gallium sensitive isolate would display comparable copper sensitivity. We have demonstrated that this was not the case since variable sensitivities were observed.

Intraspecies variability may be a result of a number of factors, including the presence of inherent or acquired metal resistant elements [50]. Some of these mechanisms include toxin export, reduced uptake [37] and changes to the extracellular biofilm [32]. The source of an isolate, such as an antibiotic exposed wound versus the lungs of a cystic fibrosis patient, likely plays a large role in mediating the aforementioned factors. For example, isolates obtained from a burn wound undergoing treatment are conditioned and therefore present elevated resistance when compared to those obtained elsewhere, such as a urine sample. Studies have demonstrated that agents other than antibiotics, such as metals, can select for antibiotic resistance [46,51,52] and the opposite may also hold true. As a result, isolates obtained from the wound sample may demonstrate greater metal resistance, regardless of the species, since it has undergone selective pressures that permit the expression of antimicrobial resistant factors [53]. Furthermore, if an isolate was extracted from a multispecies consortium, horizontal gene transfers and changes in the metabolic profile of an organism may have large effects on the susceptibility of an organism to an external challenge [54]. For instance, our group has shown that in the presence of metals, a dual-species biofilm composed of *P. aeruginosa* and *S*. *aureus* demonstrates elevated resistance when compared to a single species biofilm and other microbe combinations [53].

Differences among interspecies susceptibility may be a result of the same factors that influence intraspecies variability, including genetic differences, thereby causing alterations in the proteomic and metabolic profile of the organism. Additional influences are likely to exist, such as differences in the LPS (or lack thereof in the case of Gram-positive organisms); substantial changes in the surrounding biofilm, its constituents and the biomolecules exerted by the organism [37]; and varying ratios of lipids. For example, whereas both *E*. *coli* and *P. aeruginosa* are Gram-negative bacteria, inner membrane lipid ratios differ. Within the membrane of *P. aeruginosa* and not *E*. *coli*, three additional lipids are found as the foremost components, including phosphatidylcholine ornithine lipid and alanyl-phosphatidylglycerol [55].

Finally, to account for differences in the metal concentrations, the isolate scores were normalized against the working stock solutions (Supplementary Materials, Figure S2). Our data conveys that if the working concentration of silver increased from 0.5 M to 1.0 M, 100% of the isolates would have sensitive profiles, as observed in Supplementary Materials, Figure S2. This concentration would guarantee the prevention of the growth of the organism tested under the conditions used. To no surprise, we determined that silver was the most efficacious metal. The utilization of this metal for both commercial and medical use is far greater than the remaining metals [56,57,58] for valid reasons. Copper is also finding its way into healthcare settings and is currently being used for commercial purposes [59]. Nonetheless, under the concentration of copper used in this study, only approximately 50% of the microorganisms tested were marked as sensitive. We conclude that whilst it is still a useful metal antimicrobial, greater care must be taken when using this metal in comparison to silver. Nickel was found to be the least effective metal and not surprisingly since the use of nickel as an antimicrobial is not acclaimed [13,60]. This is likely due to the efficient and regulated uptake, trafficking and storage of this metal [61]. Copper, which is also tightly regulated in the cell, displays higher binding affinity to biomolecules, based on the Irving-Williams series [62], when compared to nickel and zinc. In fact, intracellular metal concentrations are generally inversely correlated with Irving-Williams series in that a greater binding affinity of a cation results in lower intracellular concentration and subsequently greater toxicity when present.

In this work, we asked whether the efficacies of metal antimicrobials were comparable between species and amongst isolates of the same species. Using the standard method of testing, namely the disk diffusion assay, we were able to validate that the species respond dissimilarly to metal stress and isolates of the same species display different metal susceptibilities. Despite the perception that metals are broad-spectrum antimicrobials, certain metals perform better against particular isolates and species. In summary, great care must be taken when using metal-based antimicrobials both in healthcare, industrial and consumer settings due to their variable efficacies.

## 4. Materials and Methods

### 4.1. Bacterial Strains and Storage

All organisms, including those identified as strains, such as *P. aeruginosa* PAO1, are referred to as isolates in this work for ease. The strains, namely *S. aureus* ATCC 25923, *P. aeruginosa* ATCC 27853 and *E. coli* ATCC 25922 are distinguished as indicator strains, which was noted by the American Type Culture Collection.

The *Pseudomonas aeruginosa* isolates and uropathogenic *Escherichia coli* CFTO73 were generous gifts from Dr. J. Harrison (University of Calgary). All bacterial stains and isolates were stored in Microbank™ vials at −80 °C as described by the manufacturer (ProLab Diagnostics, Richmond Hill, ON, Canada). Prior to the disk diffusion assay, the strains and isolates were streaked out on Luria-Bertani (LB) media agar (1.5%) plates and grown overnight at 37 °C. Our choice of growth medium is reflected in other works that have also used this medium to monitor the susceptibility of microorganisms to metals.

### 4.2. Determination of the Effective Metal Concentrations and Metal Storage

The minimal planktonic bactericidal concentrations of the indicator strains, namely *S. aureus* ATCC 25923, *P. aeruginosa* ATCC 27853 and *E. coli* ATCC 25922, were determined in a previous report by Gugala et al. [13]. These concentrations, which were determined under identical conditions as in this study, were used as a means of normalizing and providing context to the zones of growth inhibition. 

Silver nitrate (AgNO_3_), copper sulfate (CuSO_4_), gallium nitrate [Ga(NO_3_)_3_] and nickel sulfate (NiSO_4_·6H_2_O) were obtained from Sigma-Aldrich (St. Louis, MO, USA). Aluminum sulfate [Al_2_(SO_4_)_3_·H_2_O] was obtained from Matheson Colman and Bell (Norwood, OH, USA) and zinc sulfate (ZnSO_4_·7H_2_O) was obtained from Fisher Scientific (Fair Lawn, NJ, USA). The working stock solutions of each metal are as follows: silver nitrate—0.5 M, copper sulfate—2.0 M, gallium nitrate—1.0 M, nickel sulfate—2.5 M, aluminum sulfate—1.0 M and zinc sulfate—1.5 M. All stock solutions were stored in distilled and deionized water (dd)H_2_O at 21 °C. Finally, to ensure that microbial growth was not impeded by the accompanying counter ions, the stock solutions of sodium nitrate (NaNO_3_) and sodium sulfate (Na_2_SO_4_), with a concentration of 1.5 M and 2.5 M, respectively, were made, tested and stored for no longer than two weeks in (dd)H_2_O at 21 °C. Neither the blank disks nor the counterion loaded disks were found to influence the measured zones of growth inhibition.

### 4.3. Bacterial Growth and the Agar Disk-Diffusion Method

All chemicals were obtained from VWR international, Mississauga, Canada. Bacterial growth and susceptibility testing using the disk diffusion assay followed the Clinical and Laboratory Standards Institute’s guidelines for bacterial testing [63]. Firstly, the bacterial isolates were grown for 16 hours in filter-sterilized chemically simulated wound fluid (CSWF) modified from Werthén et al. [64] (50% peptone water (0.85% NaCl, 0.1 g/L peptone): 50% bovine serum albumin (66 g/L)]. Mueller Hinton media is commonly the medium used for disk diffusion assays. Despite this, we predicted that the supplemented acid hydrolysate of casein may lead to increased metal chelation due to the high level of amino acids found in this ingredient. In other works, amino acids are used as a means of sequestering metal ions when performing susceptibility testing [13,53,65]. Therefore, the rich medium, CSWF, was selected as it closely mimics a wound environment. The following day, sterile 6 mm filter disks were soaked in each metal for 30 minutes. Any remaining metal solution was removed to ensure that the disks were not oversaturated. Moreover, to prevent crystallization, the disks were not permitted to dry. After this, 250 μL of inoculum, standardized to an optical density of 1.00 (A_600_), was added onto fresh LB agar (1.5%) plates, spread uniformly and allowed to dry. The metal loaded and control disks were placed on the plates and incubated overnight at 37 °C. The following day, the zones of growth inhibition were measured to the nearest millimeter.

Each biological trial included two technical replicates and the indicator strain corresponding to the isolate tested. In total, three biological trials were completed, with a total of six replicates.

### 4.4. Normalization and Statistical Analyses

As aforementioned, the indicator strains, namely *E*. *coli* ATCC 25922, *P*. *aeruginosa* ATCC 27853 and *S*. *aureus* ATCC 25923, were included as a means of normalizing and providing context to the zones of growth inhibition. This was largely due to the variability between trials and the lack of clinical breakpoint information for the given strains under metal ion challenge.

First, the technical replicates within the same biological trial were averaged and these means were used for subsequent analyses. After this, working within a biological trial, the means of the isolates were normalized against the mean of reference indicator strain and finally, the scores of each isolate under a given metal challenge were averaged. Below is an example for one isolate normalized against the values of the indicator strains, with each having two technical replicates in the same biological trial:

*E. coli* ATCC 25922 (indicator strain)

Biological trial 1 diameters for zinc (mm): 26, 27    mean: 26.5
Biological trial 2 diameters for zinc (mm): 26, 27    mean: 26.5
Biological trial 3 diameters for zinc (mm): 25, 26    mean: 25.5

*E. coli* E057

Biological trial 1 diameters for zinc (mm): 25, 25    mean: 25
Biological trial 2 diameters for zinc (mm): 25, 25    mean: 25
Biological trial 3 diameters for zinc (mm): 22. 23    mean: 22.5

Normalization:

Biological trial 1, zinc: 25/26.5 = 0.943
Biological trial 2, zinc: 25/26.5 = 0.943
Biological trial 3, zinc: 22.5/25.5 = 0.882

Final score: (0.943 + 0.943 + 0.882)/3 = 0.923 

A score of 1.0 indicated no difference in susceptibility when compared to the indicator strain. Isolates with scores <1.0 were noted as resistant since the zones of growth inhibition for these isolates were less than the corresponding indicator strain. Those with scores >1.0 were regarded as sensitive since the zones of growth inhibition were larger than the indicator strain. Furthermore, since the MICs of the indicator strains were known and these strains were used to normalize the dataset, a qualitative concentration—referred to as the breakpoint value—can be attributed to each isolate. As a result, a score of 1.0 is equal to the MIC of the reference indicator strain under the given metal stress. A score <1.0 means that the breakpoint value is >MIC and a score >1.0 means that the breakpoint value is <MIC.

Finally, to account for the different metal concentrations used, the scores were normalized against the working stock solutions, which subsequently disclosed the most efficacious metal. Here, the efficacies are only compared between each metal and the breakpoint value is no longer applicable. This normalization is based on the assumption that the metals diffuse through the agar equivalently.

## Supplementary Materials

The following are available online at https://www.mdpi.com/2079-6382/8/2/51/s1, Table S1: *Escherichia coli* zones of growth inhibition (mm) and corresponding breakpoint values, Table S2: *Pseudomonas aeruginosa* zones of growth inhibition (mm) and corresponding breakpoint values, Table S3: *Staphylococcus aureus* zones of growth inhibition (mm) and corresponding breakpoint values. Figure S1: Heatmap signifying the normalized zones of growth inhibition, Figure S2: Heatmap representing the zones of growth inhibition normalized against the concentration of metal.

## Appendix A

**Table A1:** Minimal inhibitory concentrations previously determined by Gugala et al. in chemically simulated wound fluid [13].

Metal	*Escherichia coli* ATCC 25922	*P. aeruginosa* ATCC 27853	*S. aureus* ATCC 25923
Aluminum [Al_2_(SO_4_)_3_·H_2_O]	250 mM	1.95 mM	> 250 mM
Copper (CuSO_4_)	12.5 mM	6.25 mM	12.5 mM
Gallium [Ga(NO_3_)_3_·H_2_O]	31.25 mM	15.63 mM	15.63 mM
Nickel (NiSO_4_)	>625 mM	>625 mM	>625 mM
Silver (AgNO_3_)	>0.50 mM ^1^	>0.50 mM ^1^	>0.50 mM ^1^
Zinc (ZnSO_4_·7H_2_O)	>650 mM	> 375 mM ^1^	23.44 mM

^1^ Maximum concentration of silver reached before significant precipitation occurred, see [13] for more information. This observation was also demonstrated to be organism-specific since the MIC were found for some organisms but not for others under the same metal antimicrobial.

## References

[1] Davies J., Davies D. (2010). Origins and Evolution of Antibiotic Resistance. Microbiol. Mol. Biol. Rev..

[2] Neu H.C. (1993). The Crisis in Antibiotic Resistance. Science.

[3] Park A.J., Okhovat J.P., Kim J. (2017). Antimicrobial Peptides. Clin. Basic Immunodermatol..

[4] Kenawy E.R., Worley S.D., Broughton R. (2007). The Chemistry and Applications of Antimicrobial Polymers: A State-of-the-Art Review. Biomacromolecules.

[5] Tyers M., Brown E.D., Wildenhain J., Farha M.A., Wright G.D., Coombes B.K., Ejim L., Falconer S.B. (2011). Combinations of Antibiotics and Nonantibiotic Drugs Enhance Antimicrobial Efficacy. Nat. Chem. Biol..

[6] Turner R.J. (2017). Metal-Based Antimicrobial Strategies. Microb. Biotechnol..

[7] Dupont C.L., Yang S., Palenik B., Bourne P.E. (2006). Modern Proteomes Contain Putative Imprints of Ancient Shifts in Trace Metal Geochemistry. Proc. Natl. Acad. Sci. USA.

[8] Waldron K.J., Robinson N.J. (2009). How Do Bacterial Cells Ensure That Metalloproteins Get the Correct Metal?. Nat. Rev. Microbiol..

[9] Andreini C., Bertini I., Rosato A. (2004). A Hint to Search for Metalloproteins in Gene Banks. Bioinformatics.

[10] Nies D.H. (1999). Microbial Heavy-Metal Resistance. Appl. Microbiol. Biotechnol..

[11] Harrison J.J., Ceri H., Stremick C.A., Turner R.J. (2004). Biofilm Susceptibility to Metal Toxicity. Environ. Microbiol..

[12] Kaneko Y., Thoendel M., Olakanmi O., Britigan B.E., Singh P.K. (2007). The Transition Metal Gallium Disrupts *Pseudomonas aeruginosa* Iron Metabolism and Has Antimicrobial and Antibiofilm Activity. J. Clin. Invest..

[13] Gugala N., Lemire J.A., Turner R.J. (2017). The Efficacy of Different Anti-Microbial Metals at Preventing the Formation of and Eradicating Bacterial Biofilms of Pathogenic Indicator Strains. J. Antibiot..

[14] Harrison J.J., Turner R.J., Ceri H. (2005). High-Throughput Metal Susceptibility Testing of Microbial Biofilms. BMC Microbiol..

[15] Khan S.T., Musarrat J., Al-Khedhairy A.A. (2016). Countering Drug Resistance, Infectious Diseases and Sepsis Using Metal and Metal Oxides Nanoparticles: Current Status. Colloids Surf. B Biointerfaces.

[16] Wright J.B., Lam K., Burrell R.E. (1998). Wound Management in an Era of Increasing Bacterial Antibiotic Resistance: A Role for Topical Silver Treatment. Am. J. Infect. Control.

[17] Mikolay A., Huggett S., Tikana L., Grass G., Braun J., Nies D.H. (2010). Survival of Bacteria on Metallic Copper Surfaces in a Hospital Trial. Appl. Microbiol. Biotechnol..

[18] Palza H., Nuñez M., Bastías R., Delgado K. (2018). In Situ Antimicrobial Behavior of Materials with Copper-Based Additives in a Hospital Environment. Int. J. Antimicrob. Agents.

[19] Shastri J.P., Rupani M.G., Jain R.L. (2012). Antimicrobial Activity of Nanosilver-Coated Socks Fabrics against Foot Pathogens. J. Text. Inst..

[20] Atiyeh B.S., Costagliola M., Hayek S.N., Dibo S.A. (2007). Effect of Silver on Burn Wound Infection Control and Healing: Review of the Literature. Burns.

[21] Borkow G., Zatcoff R.C., Gabbay J. (2009). Reducing the Risk of Skin Pathologies in Diabetics by Using Copper Impregnated Socks. Med. Hypotheses.

[22] Li Y., Leung P., Yao L., Song Q.W., Newton E. (2006). Antimicrobial Effect of Surgical Masks Coated with Nanoparticles. J. Hosp. Infect..

[23] Borkow G., Zhou S.S., Page T., Gabbay J. (2010). A Novel Anti-Influenza Copper Oxide Containing Respiratory Face Mask. PLoS ONE.

[24] Ghazvini K., Barati S., Ahrari F., Eslami N., Rajabi O. (2015). The Antimicrobial Sensitivity of *Streptococcus mutans* and *Streptococcus sangius* to Colloidal Solutions of Different Nanoparticles Applied as Mouthwashes. Dent. Res. J. (Isfahan)..

[25] Liu Y., He L., Mustapha A., Li H., Hu Z.Q., Lin M. (2009). Antibacterial Activities of Zinc Oxide Nanoparticles against *Escherichia coli* O157:H7. J. Appl. Microbiol..

[26] Hernández-Sierra J.F., Ruiz F., Cruz Pena D.C., Martínez-Gutiérrez F., Martínez A.E., de Jesús Pozos Guillén A., Tapia-Pérez H., Martínez Castañón G. (2008). The Antimicrobial Sensitivity of *Streptococcus mutans* to Nanoparticles of Silver, Zinc Oxide and Gold. Nanomed. Nanotechnol. Biol. Med..

[27] Radzig M.A., Nadtochenko V.A., Koksharova O.A., Kiwi J., Lipasova V.A., Khmel I.A. (2013). Antibacterial Effects of Silver Nanoparticles on Gram-Negative Bacteria: Influence on the Growth and Biofilms Formation, Mechanisms of Action. Colloids Surfaces B Biointerfaces.

[28] Wahid F., Zhong C., Wang H., Hu X., Chu L. (2017). Recent Advances in Antimicrobial Hydrogels Containing Metal Ions and Metals/Metal Oxide Nanoparticles. Polymers (Basel)..

[29] Palza H. (2015). Antimicrobial Polymers with Metal Nanoparticles. Int. J. Mol. Sci..

[30] Li P., Li J., Wu C., Wu Q., Li J. (2005). Synergistic Antibacterial Effects of Beta-Lactam Antibiotic Combined with Silver Nanoparticles. Nanotechnology.

[31] Fayaz A.M., Balaji K., Girilal M., Yadav R., Kalaichelvan P.T., Venketesan R. (2010). Biogenic Synthesis of Silver Nanoparticles and Their Synergistic Effect with Antibiotics: A Study against Gram-positive and Gram-negative Bacteria. Nanomed. Nanotechnol. Biol. Med..

[32] Lemire J.A., Harrison J.J., Turner R.J. (2013). Antimicrobial Activity of Metals: Mechanisms, Molecular Targets and Applications. Nat. Rev. Microbiol..

[33] Balouiri M., Sadiki M., Ibnsouda S.K. (2016). Methods for in Vitro Evaluating Antimicrobial Activity: A Review. J. Pharm. Anal..

[34] Azam A. (2012). Antimicrobial Activity of Metal Oxide Nanoparticles against Gram-positive and Gram-negative Bacteria: A Comparative Study. Int. J. Nanomedicine.

[35] Ruparelia J.P., Kumar A., Duttagupta S.P. (2008). Strain Specificity in Antimicrobial Activity of Silver and Copper Nanoparticles. Acta Biomater..

[36] Vance M.E., Kuiken T., Vejerano E.P., McGinnis S.P., Hochella M.F., Hull D.R. (2015). Nanotechnology in the Real World: Redeveloping the Nanomaterial Consumer Products Inventory. Beilstein J. Nanotechnol..

[37] Harrison J.J., Ceri H., Turner R.J. (2007). Multimetal Resistance and Tolerance in Microbial Biofilms. Nat. Rev. Microbiol..

[38] Foster T.J. (1983). Plasmid-Determined Resistance to Antimicrobial Drugs and Toxic Metal Ions in Bacteria. Microbiol. Rev..

[39] Teitzel G.M., Parsek M.R. (2003). Heavy Metal Resistance of Biofilm and Planktonic *Pseudomonas aeruginosa*. Appl. Environ. Microbiol..

[40] Peeters E., Nelis H.J., Coenye T. (2008). Resistance of Planktonic and Biofilm-Grown *Burkholderia cepacia* Complex Isolates to the Transition Metal Gallium. J. Antimicrob. Chemother..

[41] Nies D.H. (2003). Efflux-Mediated Heavy Metal Resistance in Prokaryotes. FEMS Microbiol. Rev..

[42] Hobman J.L., Crossman L.C. (2015). Bacterial Antimicrobial Metal Ion Resistance. J. Med. Microbiol..

[43] Harrison J.J., Rabiei M., Turner R.J., Badry E.A., Sproule K.M., Ceri H. (2006). Metal Resistance in *Candida* Biofilms. FEMS Microbiol. Ecol..

[44] Han D., Hur H. (2018). Metagenomic analysis reveals the prevalence and persistence of antibiotic-and heavy metal-resistance genes in wastewater treatment plant. J. Microbiol..

[45] Li A.-D., Li L.-G., Zhang T. (2015). Exploring Antibiotic Resistance Genes and Metal Resistance Genes in Plasmid Metagenomes from Wastewater Treatment Plants. Front. Microbiol..

[46] Baker-Austin C., Wright M.S., Stepanauskas R., McArthur J.V. (2006). Co-Selection of Antibiotic and Metal Resistance. Trends Microbiol..

[47] Wright M.S., Peltier G.L., Stepanauskas R., McArthur J.V. (2006). Bacterial Tolerances to Metals and Antibiotics in Metal-Contaminated and Reference Streams. FEMS Microbiol. Ecol..

[48] Pal C., Asiani K., Arya S., Rensing C., Stekel D.J., Larsson D.G.J., Hobman J.L. (2017). Metal Resistance and Its Association with Antibiotic Resistance. Advances in Microbial Physiology.

[49] Um M.M., Brugère H., Kérourédan M., Oswald E., Bibbal D. (2018). Antimicrobial Resistance Profiles of Enterohemorrhagic and Enteropathogenic *Escherichia coli* of Serotypes O157:H7, O26:H11, O103:H2, O111:H8, O145:H28 Compared to *Escherichia coli* Isolated from the Same Adult Cattle. Microb. Drug Resist..

[50] Blair J.M.A., Webber M.A., Baylay A.J., Ogbolu D.O., Piddock L.J.V. (2015). Molecular Mechanisms of Antibiotic Resistance. Nat. Rev. Microbiol..

[51] Seiler C., Berendonk T.U. (2012). Heavy Metal Driven Co-Selection of Antibiotic Resistance in Soil and Water Bodies Impacted by Agriculture and Aquaculture. Front. Microbiol..

[52] Chen J., Cen T., He M., Gu A.Z., Zhang Y., Li D., Li X. (2018). Sub-Inhibitory Concentrations of Heavy Metals Facilitate the Horizontal Transfer of Plasmid-Mediated Antibiotic Resistance Genes in Water Environment. Environ. Pollut..

[53] Lemire J.A., Kalan L., Gugala N., Bradu A., Turner R.J. (2017). Silver Oxynitrate–an Efficacious Compound for the Prevention and Eradication of Dual-Species Biofilms. Biofouling.

[54] Burmølle M., Webb J.S., Rao D., Hansen L.H., Sørensen S.J., Kjelleberg S. (2006). Enhanced Biofilm Formation and Increased Resistance to Antimicrobial Agents and Bacterial Invasion Are Caused by Synergistic Interactions in Multispecies Biofilms. Appl. Environ. Microbiol..

[55] Sohlenkamp C., Geiger O. (2015). Bacterial Membrane Lipids: Diversity in Structures and Pathways. FEMS Microbiol. Rev..

[56] Politano A.D., Campbell K.T., Rosenberger L.H., Sawyer R.G. (2013). Use of Silver in the Prevention and Treatment of Infections: Silver Review. Surg. Infect. (Larchmt)..

[57] Alexander J.W. (2009). History of the Medical Use of Silver. Surg. Infect. (Larchmt)..

[58] Melaiye A., Youngs W.J. (2005). Silver and Its Application as an Antimicrobial Agent. Expert Opin. Ther. Pat..

[59] O’Gorman J., Humphreys H. (2012). Application of Copper to Prevent and Control Infection. Where Are We Now?. J. Hosp. Infect..

[60] Yasuyuki M., Kunihiro K., Kurissery S., Kanavillil N., Sato Y., Kikuchi Y. (2010). Antibacterial Properties of Nine Pure Metals: A Laboratory Study Using *Staphylococcus aureus* and *Escherichia coli*. Biofouling.

[61] Li Y., Zamble D.B. (2009). Nickel Homeostasis and Nickel Regulation: An Overview. Chem. Rev..

[62] Irving B.H., Williams R.J.P. (1953). The Stability of Transition-Metal Complexes. J. Chem. Soc..

[63] Patel J.B., Cockerill F.R., Bradford P.A., Eliopoulos G.M., Hindler J.A., Jenkins S.G., Lewis J.S., Limbago B. (2015). M02-A12: Performance Standards for Antimicrobial Disk Susceptibility Tests; Approved Standard—Twelfth Edition.

[64] Werthén M., Henriksson L., Jensen P.Ø., Sternberg C., Givskov M., Bjarnsholt T. (2010). An in Vitro Model of Bacterial Infections in Wounds and Other Soft Tissues. APMIS.

[65] Lemire J.A., Kalan L., Bradu A., Turner R.J. (2015). Silver Oxynitrate, an Unexplored Silver Compound with Antimicrobial and Antibiofilm Activity. Antimicrob. Agents Chemother..

