# Supplementary material for: Specificity in the Susceptibilities of Escherichia coli, Pseudomonas aeruginosa and Staphylococcus aureus Clinical Isolates to Six Metal Antimicrobials
Source: Antibiotics (Basel). 2019 May 1;8(2):51. doi: 10.3390/antibiotics8020051 (PMC6627307; doi:10.3390/antibiotics8020051)
Supplement: Supplementary file 1 [file antibiotics-08-00051-s001.zip › Supplementary_Antibiotics_Resubmission.docx]

Supplementary information:

Specificity in the susceptibilities of *Escherichia coli*, *Pseudomonas aeruginosa* and *Staphylococcus aureus* clinical isolates to six metal antimicrobials

Natalie Gugala ^1^, Dennis Vu^2^, Michael D. Parkins^3^ and Raymond J. Turner ^1,^*

^1^ Department of Biological Sciences, University of Calgary; ngugala@ucalgary.ca

^2^ Department of Biological Sciences, University of Calgary; [devu@ucalgary.ca](mailto:devu@ucalgary.ca)

^3^ Cumming School of Medicine, University of Calgary; mdparkin@ucalgary.ca

***** Correspondence: turnerr@ucalgary.ca; Tel.: +1 403 220 4308

Table S1: Averaged *Escherichia* *coli* zones of growth inhibition (mm), the standard deviations and the corresponding breakpoint value in the presence of the given metal, which were determined using the MIC of the indicator strain *E.* *coli* ATCC 25922 as previously observed in [13]^1,2^.

| Isolate | Aluminum | Break-  Point  value (mM) | Copper | Break-point value (mM) | Gallium | Break-point value (mM) | Nickel | Break-point value (mM) | Silver | Break-point value (mM) | Zinc | Break  Point value (mM) |
| --- | --- | --- | --- | --- | --- | --- | --- | --- | --- | --- | --- | --- |
| ATCC 25922^3^ | 17.2 ±2.3 | 250 | 19.7 ±1.2 | 125 | 13.7 ±2.8 | 31.25 | 25.0 ±2.1 | >625 | 12.6 ±2.2 | >0.50 | 25.0 ±1.4 | >650 |
| E061 | 18.1 ±0.2 | <250 | 19.4 ±0.5 | >125 | 15.4 ±0.2 | <31.25 | 22.9 ±1.2 |  | 11.9 ±1.2 |  | 21.0 ±3.2 |  |
| E056 | 16.8 ±1.8 | >250 | 19.5 ±0.4 | >125 | 14.7 ±0.4 | <31.25 | 23.3 ±2.3 |  | 10.0 ±1.1 |  | 23.5 ±0.9 |  |
| E057 | 18.1 ±1.3 | <250 | 19.8 ±1.9 | <125 | 15.0 ±1.5 | <31.25 | 25.0 ±1.1 | >625 | 11.8 ±0.6 |  | 24.2 ±1.3 |  |
| E014 | 18.3 ±1.4 | <250 | 20.5 ±0.7 | <125 | 15.6 ±0.2 | <31.25 | 24.1 ±1.2 |  | 12.1 ±0.7 |  | 23.3 ±1.1 |  |
| E060 | 16.9 ±2.7 | >250 | 20.0 ±0.4 | <125 | 15.5 ±2.8 | <31.25 | 24.4 ±0.5 |  | 12.4 ±1.6 |  | 24.0 ±0.7 |  |
| E006 | 17.0 ±2.8 | >250 | 18.8 ±1.8 | >125 | 13.5 ±2.1 | >31.25 | 22.5 ±0.7 |  | 12.4 ±0.2 |  | 22.5 ±0.0 |  |
| E047 | 17.8 ±1.8 | <250 | 19.1 ±1.2 | >125 | 15.5 ±0.7 | <31.25 | 24.3 ±0.4 |  | 12.9 ±1.2 | >0.50 | 24.3 ±0.4 |  |
| E015 | 19.1 ±0.2 | <250 | 22.4 ±0.5 | <125 | 14.4 ±1.6 | <31.25 | 25.5 ±1.4 | >625 | 12.8 ±1.8 | >0.50 | 24.1 ±0.9 |  |
| E007 | 20.0 ±0.7 | <250 | 20.0 ±0.7 | <125 | 15.4 ±0.9 | <31.25 | 24.1 ±1.2 |  | 11.4 ±0.5 |  | 23.9 ±0.2 |  |
| E022 | 18.9 ±0.5 | <250 | 20.4 ±1.2 | <125 | 13.1 ±3.7 | >31.25 | 24.0 ±0.0 |  | 11.1 ±2.3 |  | 26.0 ±1.4 | >650 |
| E045 | 18.5 ±0.7 | <250 | 21.0 ±0.7 | <125 | 12.5 ±3.5 | >31.25 | 23.9 ±0.5 |  | 11.9 ±0.2 |  | 23.6 ±0.9 |  |
| E004 | 20.0 ±0.7 | <250 | 24.3 ±0.4 | <125 | 15.3 ±0.4 | <31.25 | 27.5 ±0.7 | >625 | 14.0 ±0.7 | >0.50 | 27.1 ±0.2 | >650 |
| E029 | 19.0 ±2.8 | <250 | 20.1 ±1.9 | <125 | 19.0 ±3.0 | <31.25 | 24.8 ±2.1 |  | 12.5 ±0.7 |  | 24.5 ±2.5 |  |
| E033 | 17.1 ±0.5 | >250 | 19.1 ±0.5 | >125 | 17.4 ±1.6 | <31.25 | 22.1 ±0.2 |  | 11.4 ±1.6 |  | 23.3 ±0.4 |  |
| E012 | 18.0 ±2.5 | <250 | 19.3 ±1.4 | >125 | 15.4 ±1.6 | <31.25 | 23.3 ±2.1 |  | 10.1 ±0.9 |  | 24.0 ±1.4 |  |
| E009 | 19.8 ±1.8 | <250 | 20.8 ±1.1 | <125 | 15.0 ±0.4 | <31.25 | 23.4 ±0.2 |  | 11.6 ±0.2 |  | 23.4 ±0.9 |  |
| E005 | 15.6 ±2.3 | >250 | 18.6 ±0.2 | >125 | 13.3 ±0.4 | >31.25 | 19.3 ±2.5 |  | 11.4 ±0.2 |  | 21.8 ±1.4 |  |
| E011 | 18.6 ±2.7 | <250 | 18.8 ±0.4 | >125 | 13.0 ±1.4 | >31.25 | 23.3 ±3.2 |  | 11.5 ±2.1 |  | 23.6 ±1.9 |  |
| E112 | 18.1 ±2.7 | <250 | 18.9 ±3.3 | >125 | 13.9 ±2.7 | <31.25 | 24.4 ±1.6 |  | 13.8 ±0.0 | >0.50 | 24.3 ±0.7 |  |
| E086 | 18.6 ±0.2 | <250 | 19.5 ±0.7 | >125 | 13.9 ±0.9 | <31.25 | 23.4 ±1.6 |  | 11.8 ±2.9 |  | 23.9 ±1.9 |  |
| E109 | 17.3 ±1.1 | <250 | 21.1 ±3.0 | <125 | 12.9 ±3.3 | >31.25 | 24.4 ±0.5 |  | 12.9 ±2.3 | >0.50 | 22.6 ±0.5 |  |
| E001 | 18.1 ±3.9 | <250 | 16.5 ±2.1 | >125 | 14.3 ±1.1 | <31.25 | 22.5 ±1.1 |  | 11.8 ±1.9 |  | 23.5 ±0.7 |  |
| E105 | 17.1 ±2.3 | >250 | 20.3 ±0.4 | <125 | 13.6 ±2.7 | >31.25 | 25.4 ±0.2 | >625 | 13.9 ±1.9 | >0.50 | 24.9 ±0.5 |  |
| E092 | 16.3 ±2.1 | >250 | 19.9 ±0.5 | <125 | 11.9 ±0.5 | >31.25 | 23.8 ±1.8 |  | 13.3 ±2.8 | >0.50 | 22.9 ±0.5 |  |
| E097 | 17.3 ±2.5 | <250 | 21.9 ±0.5 | <125 | 14.4 ±1.6 | <31.25 | 24.5 ±0.7 |  | 13.6 ±2.3 | >0.50 | 24.6 ±0.2 |  |
| E100 | 16.8 ±2.5 | >250 | 19.3 ±1.1 | >125 | 13.0 ±2.1 | >31.25 | 25.1 ±0.9 | >625 | 13.8 ±0.7 | >0.50 | 25.1 ±0.2 | >650 |
| E055 | 17.9 ±2.9 | <250 | 21.8 ±3.2 | <125 | 14.4 ±3.7 | <31.25 | 26.5 ±2.8 | >625 | 13.3 ±2.5 | >0.50 | 25.5 ±1.8 | >650 |
| E096 | 19.3 ±2.5 | <250 | 22.5 ±2.5 | <125 | 14.1 ±2.7 | <31.25 | 26.5 ±0.4 | >625 | 14.4 ±0.9 | >0.50 | 26.5 ±0.7 | >650 |
| E107 | 17.9 ±2.5 | <250 | 19.8 ±2.5 | <125 | 11.9 ±2.7 | >31.25 | 25.9 ±0.4 |  | 13.6 ±0.9 | >0.50 | 24.9 ±0.7 |  |
| O157:H7^4^ | 15.1 ±2.7 | >250 | 18.0 ±0.0 | >125 | 12.8 ±0.9 | >31.25 | 22.4 ±1.9 |  | 8.8 ±1.6 |  | 23.0 ±0.2 |  |
| O127:H6 | 15.0 ±0.0 | >250 | 18.8 ±1.4 | >125 | 13.3 ±0.4 | <31.25 | 22.3 ±1.1 |  | 10.4 ±0.9 |  | 22.5 ±0.0 |  |
| CFT073 | 13.8 ±1.1 | >250 | 18.5 ±1.4 | >125 | 12.0 ±1.4 | >31.25 | 22.4 ±1.2 |  | 9.9 ±0.2 |  | 22.0 ±0.7 |  |
| F2348 | 15.0 ±0.0 | >250 | 17.6 ±0.9 | >125 | 12.9 ±0.5 | >31.25 | 22.3 ±1.8 |  | 10.8 ±1.4 |  | 23.3 ±0.4 |  |

^1^Blank indicates that the breakpoint value cannot be provided, since the precise MIC

was not determined in chemically simulated wound fluid due to metal precipitation

^2^Note that the standard deviations are calculated via the averaged replicates in order to demonstrate the variability in the data, thereby providing reasoning for our choice of normalization

^3^The MICs for the indicator strain

^4^Noted as a multi-drug resistant (MDR) bacterium [49]

Table S2: Averaged *Pseudomonas* *aeruginosa* zones of growth inhibition (mm) and the corresponding breakpoint value in the presence of the given metal, which were determined using the MIC of the indicator strain *P. aeruginosa* ATCC 27853 as previously determined by [13]^1,2^.

| Isolate | Aluminum | Break-  Point  value (mM) | Copper | Break-point value (mM) | Gallium | Break-point value (mM) | Nickel | Break-point value (mM) | Silver | Break-point value (mM) | Zinc | Break  Point value (mM) |
| --- | --- | --- | --- | --- | --- | --- | --- | --- | --- | --- | --- | --- |
| ATCC  27853^3^ | 15.0 ±0.8 | 1.95 | 16.5 ±1.3 | 6.25 | 12.7 ±1.7 | >15.63 | 21.9 ±1.6 | >625 | 13.2 ±3.2 | >0.50 | 15.9 ±1.5 | >375 |
| MM 080P03 | 15.6 ±0.9 | <1.95 | 15.5 ±1.4 | >6.25 | 18.1 ±0.9 | <15.63 | 26.6 ±1.6 |  | 17.3 ±2.5 |  | 22.8 ±3.2 |  |
| DK 122B07 | 20.1 ±0.2 | <1.95 | 19.9 ±0.5 | <6.25 | 15.1 ±1.6 | <15.63 | 33.1 ±2.7 |  | 18.0 ±1.4 |  | 19.8 ±3.2 |  |
| KS F6999 | 16.3 ±1.1 | <1.95 | 10.6 ±1.9 | >6.25 | 20.1 ±2.3 | <15.63 | 28.3 ±1.8 |  | 14.8 ±0.4 |  | 14.9 ±0.5 | >375 |
| LS 13901 | 16.8 ±0.4 | <1.95 | 18.1 ±0.5 | <6.25 | 12.5 ±1.4 | >15.63 | 28.4 ±0.2 |  | 19.5 ±0.7 |  | 19.9 ±1.9 |  |
| KR 080603 | 18.9 ± 0.5 | <1.95 | 22.1 ±1.2 | <6.25 | 26.3 ±1.8 | <15.63 | 27.6 ±2.3 |  | 16.8 ±0.4 |  | 28.5 ±2.1 |  |
| AR 091006 | 17.5 ±1.4 | 1.95 | 19.1 ±0.9 | <6.25 | 17.3 ±1.1 | <15.63 | 28.4 ±0.5 |  | 20.0 ±2.1 |  | 18.0 ±0.0 |  |
| SS 1708 | 15.8 ±1.1 | <1.95 | 12.8 ±1.8 | >6.25 | 16.6 ±0.5 | <15.63 | 23.3 ±1.1 |  | 14.0 ±1.4 |  | 19.0 ±1.4 |  |
| CD S546 | 17.5 ±0.7 | <1.95 | 19.5 ±0.7 | <6.25 | 16.0 ±0.7 | <15.63 | 29.0 ±0.0 |  | 15.0 ±2.8 |  | 21.0 ±2.1 |  |
| TB 16199 | 18.3 ±0.4 | <1.95 | 19.8 ±0.4 | <6.25 | 21.0 ±3.5 | <15.63 | 31.9 ±0.2 |  | 19.5 ±0.7 |  | 22.0 ±3.5 |  |
| PA01 | 15.8 ±0 | <1.95 | 15.3 ±0.4 | >6.25 | 15.3 ±1.8 | <15.63 | 28.8 ±3.2 |  | 17.5 ±0.7 |  | 16.9 ±0.9 |  |
| NP 104491 | 15.8 ±1.1 | <1.95 | 18.5 ±0.4 | <6.25 | 17.5 ±0.0 | <15.63 | 29.0 ±0.7 |  | 17.9 ±0.5 |  | 17.3 ±0.4 |  |
| P62 | 18.0 ±0.7 | <1.95 | 17.3 ±0.9 | <6.25 | 16.4 ±0.9 | <15.63 | 27.3 ±1.1 |  | 12.5 ±0.7 | >0.50 | 20.0 ±2.3 |  |
| P6077 | 17.0 ±1.3 | <1.95 | 18.3 ±2.0 | <6.25 | 13.1 ±3.0 | <15.63 | 23.0 ±2.5 |  | 14.4 ±1.3 |  | 17.3 ±0.4 |  |
| PCF5 | 16.8 ±3.1 | <1.95 | 20.3 ±1.1 | <6.25 | 24.2 ±2.7 | <15.63 | 29.8 ±3.8 |  | 13.1 ±2.1 | >0.50 | 27.9 ±0.8 |  |
| PMSh3 | 16.2 ±0.3 | <1.95 | 15.2 ±0.8 | >6.25 | 12.2 ±2.7 | >15.63 | 24.5 ±1.0 |  | 14.1 ±0.4 |  | 16.4 ±0.8 |  |
| PCF39s | 17.7 ±0.8 | <1.95 | 19.9 ±0.9 | <12.5 | 15.9 ±2.3 | <15.63 | 27.2 ±1.4 |  | 14.3 ±1.1 |  | 19.7 ±2.4 |  |
| PX13273 | 17.3 ±2.0 | <1.95 | 16.7 ±1.9 | <12.5 | 13.9 ±1.3 | <15.63 | 23.3 ±1.2 |  | 13.6 ±0.8 |  | 17.7 ±1.5 |  |
| PE2 | 16.7 ±0.3 | <1.95 | 17.8 ±0.3 | <12.5 | 14.6 ±0.9 | <15.63 | 25.5 ±1.0 |  | 12.0 ±1.0 | >0.50 | 17.4 ±0.4 |  |
| IM 11008 | 17.0 ±0.0 | <1.95 | 14.8 ±0.4 | >6.25 | 15.9 ±3.0 | <15.63 | 24.5 ±0.0 |  | 15.3 ±1.8 |  | 18.3 ±0.4 |  |
| LL 14837 | 18.8 ±0.4 | <1.95 | 18.5 ±0.7 | <12.5 | 14.6 ±1.6 | <15.63 | 26.3 ±3.2 |  | 14.0 ±1.1 |  | 18.0 ±1.4 |  |
| TF-F7783 | 15.3 ±0.4 | <1.95 | 16.0 ±1.4 | <12.5 | 13.1 ±2.3 | <15.63 | 23.8 ±2.5 |  | 14.3 ±0.4 |  | 20.0 ±2.8 |  |
| PT56593 | 15.3 ±0.3 | <1.95 | 15.7 ±0.3 | >6.25 | 13.2 ±1.0 | <15.63 | 23.4 ±0.4 |  | 12.5 ±0.0 | >0.50 | 17.8 ±1.5 |  |
| PT53616 | 15.8 ±1.2 | <1.95 | 16.3 ±0.7 | <12.5 | 13.8 ±1.9 | <15.63 | 20.8 ±0.7 |  | 11.8 ±2.0 | >0.50 | 17.2 ±1.8 |  |
| PCF127 | 18.8 ±1.6 | <1.95 | 19.0 ±2.5 | <12.5 | 15.5 ±1.7 | <15.63 | 27.1 ±1.7 |  | 13.7 ±0.8 |  | 18.6 ±1.4 |  |
| PS54868 | 16.3 ±1.4 | <1.95 | 15.7 ±1.0 | >6.25 | 14.6 ±2.3 | <15.63 | 22.6 ±3.9 |  | 12.6 ±0.4 | >0.50 | 16.1 ±1.8 |  |
| PJJ629 | 16.8 ±0.4 | <1.95 | 17.8 ±1.8 | <12.5 | 14.3 ±0.4 | <15.63 | 27.9 ±1.9 |  | 13.3 ±1.1 |  | 20.5 ±0.7 |  |
| PU2504 | 16.0 ±0.7 | <1.95 | 19.1 ±1.6 | <12.5 | 13.8 ±0.4 | <15.63 | 23.6 ±2.3 |  | 13.0 ±2.1 | >0.50 | 17.8 ±1.1 |  |

^1^Blank indicates that the breakpoint value cannot be provided, since the precise MIC

was not determined in chemically simulated wound fluid due to metal precipitation

^2^Note that the standard deviations are calculated via the averaged replicates in order to demonstrate the variability in the data, thereby providing reasoning for our choice of normalization

^3^The MICs for the indicator strain

**Table S3:** Averaged *Staphylococcus aureus* zones of growth inhibition (mm) and the corresponding breakpoint value in the presence of the given metal, which were determined using the MIC of the indicator strain *S. aureus* ATCC 25923 as previously observed by [13]^1,2^.

| Isolate | Aluminum | Break-point value (mM) | Copper | Break-  point value (mM) | Gallium | Break-point value (mM) | Nickel | Break-  point value (mM) | Silver | Break-point value (mM) | Zinc | Break-  point value (mM) |
| --- | --- | --- | --- | --- | --- | --- | --- | --- | --- | --- | --- | --- |
| ATCC 25923^3^ | 10.9 ±1.5 | >250 | 14.7 ±0.7 | 12.5 | 9.8 ±0.6 | 15.63 | 20.6 ±0.9 | >625 | 11.0 ±0.9 | >0.50 | 18.7 ±0.8 | 23.44 |
| MRSA362 | 12.9 ±2.7 |  | 14.9 ±1.2 | <12.5 | 9.8 ±0.4 | 15.63 | 19.4 ±2.3 |  | 10.3 ±0.3 | >0.50 | 21.5 ±0.4 | <23.44 |
| MRSA94 | 12.9 ±0.2 |  | 18.5 ±0.4 | <12.5 | 10.3 ±0.4 | <15.63 | 20.0 ±2.1 |  | 11.0 ±0.7 | >0.50 | 20.8 ±1.1 | <23.44 |
| MRSA147 | 11.6 ±0.2 |  | 14.4 ±0.9 | >12.5 | 9.8 ±0.4 | 15.63 | 21.3 ±1.1 | >625 | 10.8 ±1.1 | >0.50 | 20.6 ±0.2 | <23.44 |
| MRSA148 | 10.3 ±1.1 | >250 | 14.4 ±0.2 | >12.5 | 9.8 ±0.4 | 15.63 | 20.1 ±2.7 |  | 9.6 ±0.9 | >0.50 | 20.3 ±0.4 | <23.44 |
| MRSA166 | 11.8 ±2.5 |  | 15.1 ±1.6 | <12.5 | 9.3 ±0.0 | >15.63 | 19.5 ±1.4 |  | 10.1 ±0.9 | >0.50 | 17.9 ±0.5 | >23.44 |
| MSSA124 | 13.1 ±2.7 |  | 16.3 ±1.1 | <12.5 | 10.1 ±1.2 | <15.63 | 20.8 ±1.1 | >625 | 11.3 ±0.7 |  | 21.0 ±0.0 | <23.44 |
| MSSA103 | 12.5 ±1.4 |  | 18.3 ±1.1 | <12.5 | 11.0 ±0.7 | <15.63 | 20.9 ±0.9 | >625 | 10.8 ±0.8 | >0.50 | 19.0 ±0.0 | <23.44 |
| MSSA387 | 10.3 ±1.1 | >250 | 17.9 ±1.9 | <12.5 | 10.6 ±0.5 | <15.63 | 19.4 ±1.2 |  | 10.3 ±1.8 | >0.50 | 20.4 ±0.2 | <23.44 |
| MSRA145 | 12.0 ±2.8 |  | 16.5 ±2.8 | <12.5 | 14.0 ±2.8 | <15.63 | 16.8 ±2.8 |  | 12.0 ±1.4 |  | 18.3 ±0.4 | >23.44 |
| MRSA206 | 10.3 ±0.4 | >250 | 15.9 ±0.5 | <12.5 | 9.9 ±0.2 | <15.63 | 20.5 ±0.7 |  | 10.5 ±0.7 | >0.50 | 20.0 ±0.0 | <23.44 |
| MES26 | 14.9 ±1.6 |  | 21.3 ±0.4 | <12.5 | 13.3 ±0.4 | <15.63 | 20.9 ±0.9 | >625 | 10.0 ±0.0 | >0.50 | 29.5 ±2.8 | <23.44 |
| MES242 | 14.5 ±0.0 |  | 16.3 ±1.1 | <12.5 | 11.0 ±2.1 | <15.63 | 22.4 ±0.5 | >625 | 9.8 ±1.1 | >0.50 | 23.3 ±1.1 | <23.44 |
| MES84 | 14.8 ±0.4 |  | 21.0 ±2.1 | <12.5 | 12.5 ±1.8 | <15.63 | 23.4 ±0.9 | >625 | 11.0 ±1.4 | >0.50 | 23.9 ±0.9 | <23.44 |
| MES172 | 14.6 ±0.5 |  | 18.8 ±0.4 | <12.5 | 12.1 ±0.2 | <15.63 | 24.3 ±1.1 | >625 | 9.5 ±0.7 | >0.50 | 24.3 ±0.4 | <23.44 |
| MES116 | 14.4 ±0.5 |  | 16.8 ±0.4 | <12.5 | 8.9 ±0.2 | >15.63 | 23.0 ±0.7 | >625 | 10.9 ±0.9 | >0.50 | 23.0 ±0.7 | <23.44 |
| MRSA222 | 10.0 ±0.7 | >250 | 16.3 ±1.8 | <12.5 | 8.9 ±0.2 | >15.63 | 20.4 ±0.2 |  | 9.0 ±0.0 | >0.50 | 22.8 ±0.4 | <23.44 |
| MRSA104 | 10.5 ±0.7 | >250 | 16.5 ±0.7 | <12.5 | 9.5±0.0 | >15.63 | 21.1 ±1.2 | >625 | 9.8 ±0.7 | >0.50 | 21.8 ±0.4 | <23.44 |
| MRSA164 | 15.0 ±0.7 |  | 16.1 ±0.2 | <12.5 | 9.0 ±0.0 | >15.63 | 22.1 ±1.6 | >625 | 10.1 ±1.2 | >0.50 | 23.8 ±1.1 | <23.44 |
| MER142 | 13.8 ±0.7 |  | 18.5 ±0.2 | <12.5 | 10.0 ±0.0 | <15.63 | 23.0 ±1.6 | >625 | 10.1 ±1.2 | >0.50 | 22.8 ±1.1 | <23.44 |
| MSSA323 | 10.3 ±0.4 | >250 | 16.6 ±0.5 | <12.5 | 10.3 ±1.1 | <15.63 | 19.0 ±0.4 |  | 10.1 ±0.9 | >0.50 | 20.8 ±1.1 | <23.44 |
| MeS92 | 14.5 ±0.0 |  | 24.4 ±1.6 | <12.5 | 11.3 ±1.1 | <15.63 | 22.3 ±0.4 | >625 | 12.8 ±0.4 |  | 25.5 ±0.7 | <23.44 |
| MSSA102T | 10.0 ±0.0 | >250 | 17.4 ±1.2 | <12.5 | 10.4 ±0.2 | <15.63 | 20.0 ±0.0 |  | 9.5 ±0.7 | >0.50 | 21.1 ±0.5 | <23.44 |
| MES104K | 13.3 ±1.8 |  | 18.0 ±0.7 | <12.5 | 9.0 ±0.0 | >15.63 | 21.3 ±0.4 | >625 | 9.8 ±1.1 | >0.50 | 25.0 ±0.0 | <23.44 |
| MES103 | 14.3 ±2.5 |  | 16.0 ±0.0 | <12.5 | 9.0 ±0.0 | >15.63 | 19.5 ±0.7 |  | 10.3 ±0.4 |  | 24.8 ±0.4 | <23.44 |
| MES101T | 16.3 ±0.4 |  | 18.0 ±0.7 | <12.5 | 9.0 ±0.0 | >15.63 | 23.1 ±0.5 | >625 | 10.6 ±0.2 |  | 23.0 ±0.0 | <23.44 |
| MER257 | 11.8 ±3.2 |  | 20.0 ±7.1 | <12.5 | 11.3 ±2.5 | <15.63 | 20.8 ±3.2 |  | 9.5 ±0.0 |  | 24.0 ±2.1 | <23.44 |
| MER117 | 14.0 ±0.7 |  | 23.8 ±3.2 | <12.5 | 10.9 ±0.2 | <15.63 | 23.5 ±1.4 | >625 | 11.0 ±2.1 | >0.50 | 24.0 ±0.7 | <23.44 |
| Mer118 | 14.6 ±0.2 |  | 17.3 ±1.8 | <12.5 | 10.8 ±1.8 | <15.63 | 20.0±0.0 |  | 10.4 ±0.5 |  | 19.0 ±0.7 | <23.44 |
| Mer204 | 10.3 ±0.4 | >250 | 15.0 ±0.7 | <12.5 | 9.3 ±0.4 | >15.63 | 20.0 ±0.7 |  | 9.5 ±0.0 |  | 20.3 ±1.8 | <23.44 |
| MER212 | 14.3 ±0.4 |  | 17.3 ±0.4 | <12.5 | 9.3 ±0.4 | >15.63 | 23.0 ±0.0 | >625 | 10.0 ±0.0 |  | 25.0 ±0.7 | <23.44 |
| MER108 | 14.5 ±0.0 |  | 16.8 ±0.4 | <12.5 | 8.5 ±0.0 | >15.63 | 21.6 ±0.2 | >625 | 9.6 ±0.2 |  | 20.0 ±0.0 | <23.44 |
| MER237 | 15.6 ±0.2 |  | 21.5 ±2.1 | <12.5 | 13.5 ±0.7 | <15.63 | 23.0 ±0.7 | >625 | 10.3 ±0.4 |  | 24.0 ±0.0 | <23.44 |
| MER22 | 15.8 ±0.4 |  | 24.3 ±0.4 | <12.5 | 14.8 ±1.8 | <15.63 | 23.3 ±0.4 | >625 | 10.8 ±0.4 |  | 26.8 ±0.4 | <23.44 |
| MRSA204 | 10.8 ±0.0 | >250 | 15.8 ±2.1 | <12.5 | 10.0 ±0.0 | <15.63 | 21.8 ±0.4 | >625 | 10.6 ±0.4 |  | 19.3 ±1.1 | <23.44 |
| MER155 | 16.5 ±0.0 |  | 24.0 ±2.1 |  | 15.0 ±2.1 | <15.63 | 23.8 ±0.4 |  | 11.3 ±0.4 | >0.50 | 24.8 ±1.8 | <23.44 |

^1^Blank indicates that the breakpoint value cannot be provided, since the precise MIC

was not determined in chemically simulated wound fluid due to metal precipitation

^2^Note that the standard deviations are calculated via the averaged replicates in order to demonstrate the variability in the data, thereby providing reasoning for our choice of normalization

^3^The MICs for the indicator strain

*Escherichia*

*coli*

*Pseudomonas*

*aeruginosa*

*Staphylococcus*

*aureus*

Figure S1: Heatmap signifying the normalized zones of growth inhibition. Score of 1.0 was given to the zone of growth inhibition for the indicator strain This value also represents the MIC of that organisms under metal challenge. The isolates were normalized over the corresponding strain to yield comparable values. The color red denotes a sensitive hit (>1.0) and the color purple was given to the isolates that displayed enhanced resistant (<1.0). Data collected from the mean of three biological trials, each with two replicates; note that the working stock solutions were not equivalent. The MICs (score = 1.0) for the given data are as follows in the order: *E*. *coli* ATCC 25922, *P*. *aeruginosa* ATCC 27853 and *S*. *aureus* ATCC 25923: a) aluminum: 250 mM, 1.95 mM and >250 mM, b) copper: 12.5 mM, 6.25 mM and 12.5 mM, c) gallium: 31.25 mM, 15.63 mM, 15.62 mM, d) nickel: >625 mM, >650 mM and >625 mM, e) silver: >0.5 mM, >0.5 mM and >0.5 mM, f) zinc: >650 mM, >375 mM and 23.44 mM.

*Escherichia*

*coli*

*Pseudomonas*

*aeruginosa*

*Staphylococcus*

*aureus*

Figure S2: Heatmap representing the zones of growth inhibition normalized against the concentration of metal. Each metal was provided an equivalent score of 1.0. Red specifies isolate sensitivity therefore, it was interpreted that a concentration of 1.0 M would ensure eradication. Purple indicates resistance, hence a concentration greater than 1.0 M is required to eradicate the organism in the growth medium used in this study. Data collected from the mean of three biological trials, each with two replicates.
